# Validation studies of the Perinatal Affective Assessment Scales for Fathers (PAPA) and Mothers (PAMA) in Chilean parents

**DOI:** 10.1186/s12884-025-07747-1

**Published:** 2025-05-28

**Authors:** Francisca Pérez Cortés, Daniela Ampuero, Valentina Alba, María José Balin, Alejandra Iturra, Felipe Ayala, Michele Giannotti

**Affiliations:** 1 Adolfo Ibañez University, Psychology, Diagonal Las Torres 2640, Santiago, Peñalolén Chile; 2 San Sebastian University, Lago Panguipulli 1390, Puerto Montt, Los Lagos Chile; 3 Alberto Hurtado University, Almirante Barroso 10, Santiago, Chile; 4 eCampus University, Novedrate Como, Italy

**Keywords:** Perinatal depression, Paternal mental health, Scale validation, Perinatal affectivity

## Abstract

Perinatal depression has been extensively studied in women, but its impact on fathers remains underexplored, despite evidence showing a prevalence of around 10% in men. This study aimed to validate the Perinatal Affective Assessment Scale for Fathers (PAPA) and its maternal counterpart **(**PAMA**)** in a Chilean population, addressing the gender-specific manifestations of perinatal affective symptoms. A quantitative, cross-sectional, and correlational design was employed, including 80 fathers and 94 mothers. Confirmatory factor analysis revealed excellent fit indices for both scales. For the PAPA scale, the respecified model showed CFI = 1.000, TLI = 1.004, RMSEA = 0.000 (90% CI = 0.000–0.000), and SRMR = 0.065. For the PAMA scale, the respecified model also showed CFI = 1.000, TLI = 1.004, RMSEA = 0.000 (90% CI = 0.000–0.000), and SRMR = 0.046. Additionally, significant differences were observed between PAPA and PAMA scores, highlighting higher affective symptomatology among fathers. The results confirm the validity and reliability of the scales, emphasizing the importance of incorporating fathers into perinatal mental health evaluations from a dyadic perspective.

## Introduction

Depression is operationally defined by the Chilean Ministry of Health as a pathological alteration of mood, characterized by a decline in mood leading to sadness, accompanied by various symptoms and signs that persist for at least two weeks. Its onset affects virtually every aspect of a person’s life, such as interpersonal relationships, behavioral functioning, and cognitive functioning, being the leading cause of disability worldwide in terms of total DALYs (Disability-Adjusted Life Years) [23].

During pregnancy, depressive episodes in women have a prevalence of 7% to 15% in high-income countries and 19% to 25% in middle- and low-income countries. In the postpartum period, this prevalence approaches 10% in high-income countries and 20% in middle- and low-income countries [16]. Chile stands out with the highest reported prevalence of postpartum depression among the 56 countries that collect data on this condition [19], estimating that between 16 and 24% experience it within the first six months postpartum [12, 29]. Perinatal depression (both pre- and postpartum) has various negative consequences, such as a higher prevalence of risk behaviors in mothers, disruptions in mother-infant interaction, and problems in the cognitive and socio-emotional development of the child [7, 18]. Given the relevance of maternal mental health during the perinatal period, the Chilean Ministry of Health has issued guidelines for the early detection and treatment of depression during this stage. Screening for depressive symptoms using the Edinburgh Postnatal Depression Scale (EPDS) reaches 89% of pregnant women nationwide [29].

Although health policies are gradually approaching a systemic and family-oriented understanding of mental health, paternal mental health assessments are still not included. This omission persists despite evidence indicating that during the perinatal period, the emotional states of mothers and fathers influence each other, showing significant correlations between depressive disorders [31, 34]. Paternal perinatal depressive symptoms also impact family life, with evidence suggesting negative effects on the emotional, behavioral, and psychomotor development of infants and children, family dynamics, and marital satisfaction [4, 5, 28, 33].

This suggests that exclusive evaluation of the mother is insufficient and stems from cultural premises rather than scientific data or research. Although no epidemiological studies evaluate paternal depressive symptoms in Chile, exploratory research found that nearly 20% of men exhibit symptoms within the first months after the birth of a child [26].

### Gender differences in the manifestation of symptoms and the evaluation of perinatal affectivity

In assessing paternal affectivity, it is essential to consider that depressive symptoms in men may often manifest differently than in women. Due to psychosocial influences, men tend to express their emotional distress through externalizing and behavioral symptoms rather than through typically depressive responses [5, 30]. In fact, compared to maternal perinatal depression, paternal depression can co-occur with other disorders whose symptoms may overlap or mask it [5, 9, 17, 25].

A study conducted in Chile on the subjective meaning men attribute to fatherhood and depression found that gender-related difficulties hinder the detection of depressive symptoms. For men, depression is often associated only with its most extreme expressions and is perceived as contrary to cultural notions of fatherhood, masculinity, and the role of provider. This cultural framework makes recognizing depressive symptoms a challenging and even threatening process for men [24].

These gender-related qualitative differences in the expression of depressive symptoms mean that instruments designed for mothers lose sensitivity when applied to male samples. The closest alternative is the paternal validation of the Edinburgh Postnatal Depression Scale (EPDS), which was originally developed for mothers. Although the EPDS has demonstrated high sensitivity with a cutoff score of 12, it has been suggested that it may not adequately capture mild depression, anxiety disorders and other affective clinical manifestations [3, 10, 22].

### The PAPA and PAMA scales

The Perinatal Affective Assessment Scale for Fathers (PAPA), the questionnaire used in this study was originally developed for the Italian population, it is a straightforward screening tool for men that has demonstrated adequate validity and internal consistency [2, 6]. It is the first self-reported questionnaire specifically designed for fathers to early detect perinatal affective symptoms. This study seeks to advance the validation of the PAPA and its maternal counterpart, the Perinatal Affective Assessment Scale for Mothers (PAMA), in Chile. The goal is to provide gender-sensitive tools for early detection of depressive/affective symptoms and to further understand this phenomenon from a dyadic perspective, acknowledging the mutual influence of depressive symptoms between fathers and mothers. Validating this instrument will enable the initiation of paternal mental health screening in Chile’s primary healthcare system during the prenatal period.

## Methodology

### Participants

The sample consisted of 80 men and 94 women. The average age of the men was 35.8 years (SD = 5.56, range = 23–48), while the average age of the women was 33.7 years (SD = 4.40, range = 21–44). Full sociodemographic characteristics are summarized in Table 1.

**Table 1:** Sociodemographic characteristics of the sample

Variable	Men (n = 80)	Women (n = 94)
**Age (years)**	35.8 (SD = 5.56)	33.7 (SD = 4.40)
**Educational Level**		
High School	7.5%	3.3%
Technical-Superior	3.75%	5.4%
University	12.5%	47.8%
Postgraduate	33.75%	30.4%
**Socioeconomic status**		
Low	4.9%	4.4%
Lower-Middle	0%	4.4%
Middle	8.5%	2.9%
Upper-Middle	12.2%	14.7%
Upper	65.9%	69.1%
No specified	8.5%	4.4%
**Residence**		
Metropolitan Region	70%	58.7%
Other Regions	Valparaíso, Magallanes, Los Lagos	Valparaíso, Magallanes, Los Lagos
**Marital Status**		
Married	55%	43.48%
Single	13.75%	18.48%
Cohabiting	27.5%	36.96%
Separated/Divorced	3.75%	1.09%
**Family Structure**		
Couple without children	40.3%	46.7%
Couple with child/children	51.6%	28.3%
Monoparental with child/children	0%	5%
Extended families	22%	11.7%
Multigenerational household	6.5%	5%
Stepchildren or non-biological figures	3.2%	3.3%
**Number of Childrens**		
0	31%	35.1%
1	44%	45.6%
2	15%	14%
Más de 2	10%	5.3%

The data represent percentages for each subcategory of men and women within the total sample

### Procedure

The study invited men whose partners were pregnant and women in gestation. Participants were recruited through key informants, social media, and snowball sampling. Inclusion criteria included being of legal age, residing in Chile, and being in the second or third trimester of pregnancy. Exclusion criteria included high-risk pregnancies, severe health issues, or a history of psychiatric disorders. To increase participation and achieve a larger sample size, instruments were administered online via Google Forms, allowing participants from across the country to respond remotely, requiring only internet access.

All participants provided signed informed consent, approved by the Ethics Committee of the Universidad Adolfo Ibañez, which explained the purpose of the study and its voluntary nature. Collected data were treated confidentially under Chilean law 19.628 on the protection of private life and personal data. Upon completion of the study, participants received a report of their results, and those presenting perinatal depressive symptoms were referred to appropriate healthcare services.

### Instruments

#### Sociodemographic questionnaire

A custom-designed tool to gather specific sociodemographic information (age, marital status, number of children, family characteristics and origin, cultural and social background, etc.).

#### Perinatal Affective Assessment Scales (PAPA and PAMA)

Self-report questionnaires developed by Baldoni, Matthey, Agostini, Schimmenti, and Caretti [6] to assess various psychosocial and physical dimensions related to perinatal affectivity experienced by a parent during pregnancy and up to one year postpartum [6]. Each scale consists of 11 items describing issues rated by severity ("Not at all,""A little,""Moderately,""A lot") or as"Yes,""Possibly,"or"No."Dimensions evaluated include anxiety, depression, perceived stress, irritability/anger, relationship issues, somatization, physiological problems, addictions, and other risk behaviors. The final three items address parenthood, motherhood, and cultural factors. The Perinatal Assessment of Paternal Affectivity (PAPA) and of Maternal Affectivity showed adequate psychometric properties [2, 4]. Reliability analysis of the PAPA indicated good internal consistency (McDonald’s ω = 0.860, ordinal α = 0.852) and moderate test–retest reliability over three months (ICC = 0.59). Concurrent validity was supported by significant correlations with depression (CES-D, ρ = 0.44), psychological distress (GSI, ρ = 0.67), and perceived stress (PSS, ρ = 0.51). Regarding the PAMA, internal consistency reliability was acceptable, with McDonald’s ω = 0.76 and ordinal α = 0.78. Significant positive correlations were found with depression (CES-D, ρ = 0.54), global severity of psychological symptoms (GSI, ρ = 0.61), and perceived stress (PSS, ρ = 0.57), confirming its concurrent validity. It is worth noting that the reliability indices reported above refer to previous validation studies [2, 4]. Reliability indices derived from the current sample are presented in the Results sectionClick or tap here to enter text.

#### CES-D (Center for Epidemiologic Studies Depression Scale)

A 20-item self-report scale developed for epidemiological studies of depressive symptoms [27]. It distinguishes between depressed individuals and control groups, with a five-minute completion time. In the present sample, internal consistency was acceptable (Cronbach’s α = 0.71), consistent with previous studies [4, 27]. It distinguishes between depressed individuals and control groups, with a five-minute completion time.

#### SCL-90-R (Symptom Checklist-90-Revised)

We used the Symptom Checklist-90-Revised (SCL-90-R; [13]), a 90-item self-report questionnaire designed to assess a wide range of psychological symptoms. It includes nine subscales and three global indices: the Global Severity Index (GSI), the Positive Symptom Distress Index (PSDI), and the Positive Symptom Total (PST). In the present study, we used the GSI as a global measure of psychological distress. Internal consistency for the GSI in our sample was excellent (Cronbach’s α = 0.96), in line with previous findings [2, 13].

#### PSS (Perceived Stress Scale)

Prenatal stress was assessed using the Perceived Stress Scale [11], a 10-item self-report measure that evaluates the frequency of feelings and thoughts related to stress over the past month. Items are rated on a five-point Likert scale ranging from 0 (never) to 4 (very often). Internal consistency in the present study was acceptable (Cronbach’s α = 0.75), consistent with prior literature [11] [2].

#### DAS (Dyadic Adjustment Scale)

Evaluates the quality of marital or cohabiting relationships [32]. This self-report instrument includes 32 Likert-scale items assessing dyadic satisfaction, cohesion, consensus, and affective expression. It takes five minutes to complete. In our sample, internal consistency was high (Cronbach’s α = 0.87), consistent with previous reports [2, 32].

#### EPDS (Edinburgh Postnatal Depression Scale)

A self-administered tool to detect depressive symptoms (time: five minutes). With a maximum score of 30, a score of 10 or higher indicates possible depression of varying severity. Validated in Chile for use with pregnant women, it has shown good reliability and high sensitivity [1, 21].

### Data analysis

Descriptive analyses evaluated item skewness and kurtosis using exclusion criteria of |2| and |7|, respectively [15]. To test the hypothetical unidimensional model of the PAPA and PAMA scales, a Confirmatory Factor Analysis (CFA) was conducted using the WLSMV estimator, suitable for ordinal, non-normal data [8].

Fit indices included RMSEA (≤ 0.08), CFI and TLI (≥ 0.95), and SRMR (≤ 1.0) [14, 20]. Reliability was assessed with McDonald’s ω and ordinal alpha coefficients (≥ 0.70). Concurrent validity was evaluated through Spearman correlations between the scales and external measures such as CES-D, PSS, and SCL-90-R. Analyses were performed using RStudio.

## Results

### Item analysis

Six items exhibited univariate normality, while items 2 and 8 showed significant deviations, exceeding skewness (>|2|) and kurtosis (>|7|) thresholds. This confirmed the appropriateness of the WLSMV estimator for CFAs.

### Confirmatory factor analysis

The initial model for both scales was re-specified following the analysis of modification indices, which suggested additional covariances between certain items to improve the model fit. These covariances were incorporated into the model based on theoretical and statistical criteria, resulting in improved fit indices and a better representation of the hypothetical factor structure.

**Fig. 1:**
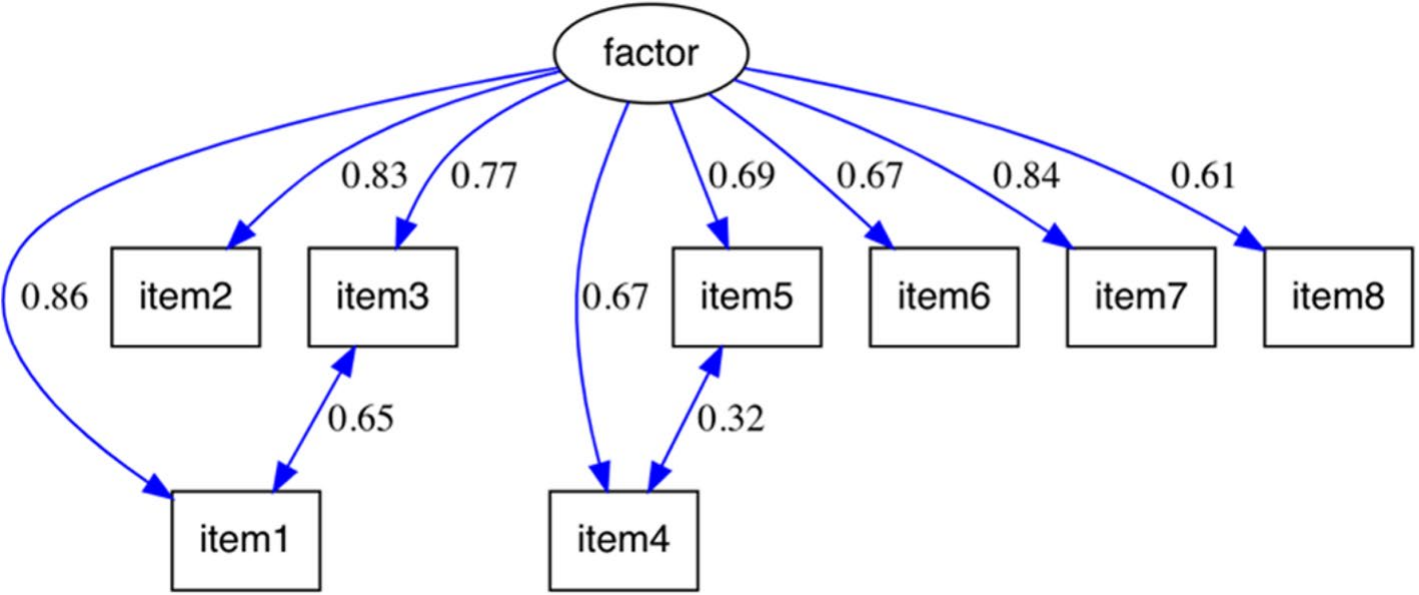
Re-specified confirmatory factor analysis model for the PAPA scale. Note: this model illustrates the relationships between the items of the PAPA scale and the proposed latent factor, including re-specified item correlations. The standardized factor loadings are displayed alongside each arrow

**Fig. 2:**
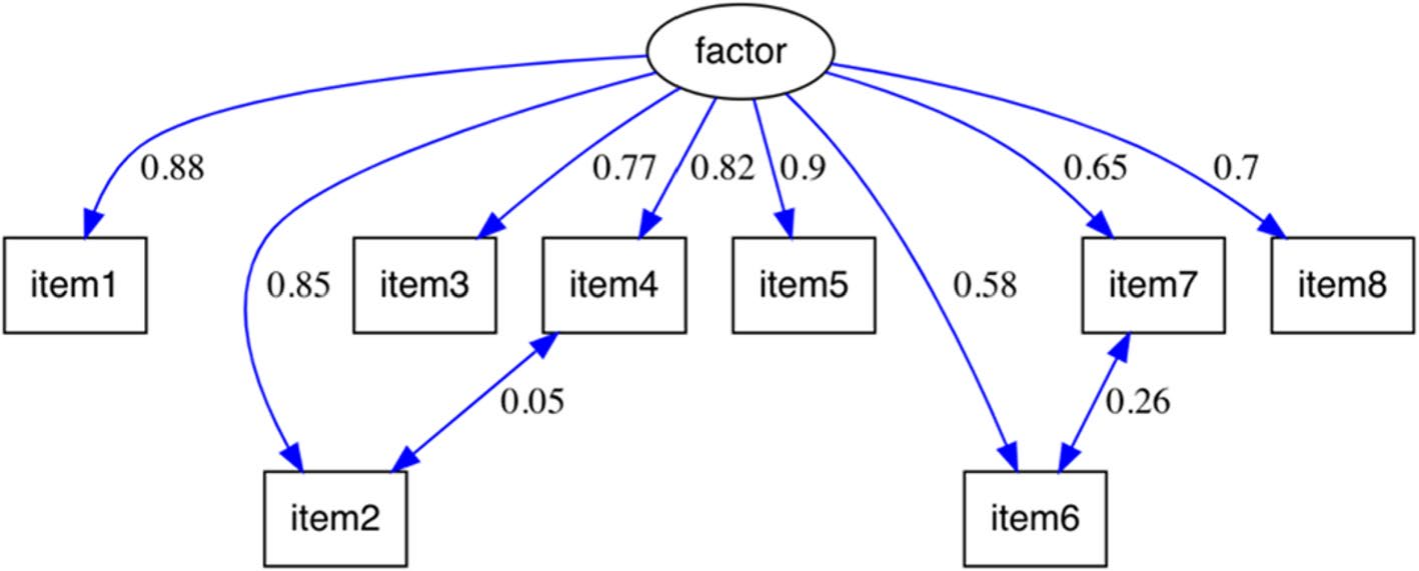
Respecified confirmatory factor analysis model for the PAMA scale. Note: this model illustrates the relationships between the items of the PAMA scale and the proposed latent factor, including the respecified correlations between items. Standardized factor loadings are presented next to each arrow

For the PAPA scale, the correlations between items and the factor loadings can be observed in Fig. 1, which represents the re-specified model for this scale. For the PAMA scale, these correlations and factor loadings are presented in Fig. 2, corresponding to its re-specified model. The model fit indices for both scales are summarized in Table 2, which highlights key indicators such as RMSEA, CFI, TLI, and SRMR, along with their respective values for the initial and re-specified models.

**Table 2:** Model fit indices for the factorial models of the PAPA and PAMA scales

Scale	Model	CFI	TLI	RMSEA (90% CI)	SRMR
PAPA	Initial	0.998	0.997	0.052 (0.000–0.115)	0.078
PAPA	Respecified	1.000	1.004	0.000 (0.000–0.000)	0.065
PAMA	Initial	1.000	1.003	0.000 (0.000–0.067)	0.052
PAMA	Respecified	1.000	1.004	0.000 (0.000–0.000)	0.046

Values represent standard indices used to assess the fit of the factorial model

*RMSEA* Root Mean Square Error of Approximation (values in parentheses represent the 90% confidence interval), *CFI* Comparative Fit Index, *TLI* Tucker-Lewis Index, *SRMR* Standardized Root Mean Square Residual

Table 2 presents the model fit indices for the factorial model of the PAPA and PAMA scales.

### Internal consistency

The internal consistency of the scales was evaluated using McDonald's ω coefficient and ordinal alpha. For the PAPA scale, McDonald's ω coefficient was 0.90, while the ordinal alpha ranged across values that included some outliers: 0.87 (average values), but with items like item 5 reaching an alpha of 6.79 and item 6 showing an alpha of 0.02. For the PAMA scale, McDonald's ω coefficient was 0.93, and the ordinal alpha showed average values of 0.89. However, outliers were also detected, such as item 5 with an alpha of 8.38 and item 6 with an alpha of 0.01.

### Concurrent validity

Concurrent validity was evaluated using Spearman correlations (ρ) between the PAPA and PAMA scales and related external measures. For the PAMA scale, high correlations were found with SCL-90 Anxiety (ρ = 0.69) and SCL-90 Depression (ρ = 0.69), as well as with SCL-90 Hostility (ρ = 0.65), supporting its ability to capture emotional dimensions. Moderate correlations were also observed with the PSS Total (ρ = 0.39) and the Global Severity Index of the SCL-90 (ρ = 0.47). In contrast, correlations with relationship dimensions measured by DAS Dyadic Cohesion were negative and low (ρ = −0.07).

For the PAPA scale, moderate correlations were found with SCL-90 Anxiety (ρ = 0.62) and SCL-90 Depression (ρ = 0.42). Correlations with PSS Total (ρ = 0.41) and SCL-90 Hostility (ρ = 0.36) were slightly lower than those observed for PAMA. Similar to PAMA, correlations with DAS Dyadic Cohesion were negative (ρ = −0.22), reflecting possible differences in how the scales evaluate dimensions related to relational functioning (Table 3).

**Table 3:** Concurrent validity correlations between the PAPA and PAMA scales and external measures

Scale	Anxiety (SCL-90, EPDS)	Depression (CES-D, SCL-90, EPDS)	Stress (PSS Total)	Irritability (Hostility)	Relationship (DAS)	Abnormality (Somatization)	Physiological (SCL-90)
PAMA	0.69, 0.55	0.36, 0.69, 0.69	0.39	0.65	0.19, −0.07, −0.34	0.62	0.47, 0.45, 0.44
PAPA	0.62, 0.54	0.33, 0.42, 0.45	0.41	0.36	0.34, −0.22, −0.28	0.54	0.42, 0.42, 0.35

Correlations are presented as ρ (Spearman). External measures include: Anxiety (SCL-90, EPDS), Depression (CES-D, SCL-90, EPDS), Perceived Stress (PSS Total), Irritability/Anger (SCL-90 Hostility), Relationship (DAS: Satisfaction, Cohesion, Dyadic Consensus), Abnormal Behavior (SCL-90 Somatization), and Physiological Problems (SCL-90 Global Severity Index, Positive Symptoms, Total Positive Symptoms)

### Differences between the scales

The differences between the two scales were analyzed using Student's t-tests, showing significant discrepancies in several items and in the total scores (p < 0.05). The results indicate that the average item scores on the PAPA scale are consistently higher than those on the PAMA scale, suggesting greater perinatal affective symptomatology reported by fathers compared to mothers (Table 4).

**Table 4:** Results of student's t-tests for the PAPA and PAMA scales

Item	Mean PAPA	Mean PAMA	Mean Difference	Confidence Interval (95%)	t-statistic	*p*-value
1	2.01	1.43	0.58	[0.30, 0.88]	3.98	0.0001
2	1.40	0.88	0.52	[0.26, 0.77]	3.96	0.0001
3	2.06	1.16	0.90	[0.60, 1.21]	5.81	< 0.0001
4	1.49	0.98	0.51	[0.23, 0.79]	3.56	0.0005
5	1.23	0.67	0.56	[0.33, 0.78]	4.97	< 0.0001
6	1.69	1.28	0.41	[0.11, 0.71]	2.72	0.0073
7	1.69	1.40	0.28	[0.01, 0.56]	2.02	0.0447
8	1.46	0.36	1.10	[0.87, 1.33]	9.41	< 0.0001
**Total**	**14.19**	**9.39**	**4.80**	**[3.14, 6.45]**	**5.72**	**< 0.0001**

The table summarizes the results of Student's t-tests for each item and the total scores between the PAPA and PAMA scales. Significant differences (*p* < 0.05) indicate variations in the scales'sensitivity to measure the evaluated constructs

## Discussion

The findings of this study provide strong evidence of the validity and reliability of the PAPA and PAMA scales for assessing psychological constructs in clinical contexts. Confirmatory Factor Analyses (CFA) demonstrated excellent fit for both unidimensional models, with metrics such as CFI and TLI consistently exceeding 0.99, and RMSEA and SRMR within acceptable ranges or very close to them. Standardized factor loadings were significant for most items, with the exception of item 9 on the PAMA scale, which showed a weaker contribution to the overall construct. This item aims to assess a high level of depressive affect through the experience of feeling profoundly miserable to the point of crying. Although it targets a clinically relevant emotional manifestation, its intense wording may have made it difficult for participants experiencing milder emotional distress to relate to it. Additionally, cultural and gender-related factors may influence individuals’ willingness to acknowledge or report crying as an expression of emotional discomfort. This could have limited response variability, thereby reducing the item's statistical weight within the model. It is therefore recommended that future applications consider revising the item’s wording to capture a broader range of depressive symptoms, while preserving its diagnostic value and ensuring greater psychometric coherence within the scale.

In terms of internal consistency, both McDonald’s ω coefficient and ordinal alpha showed high values for both scales (PAPA: ω = 0.89, PAMA: ω = 0.91), supporting their reliability. Nonetheless, outlier values were identified in certain items, specifically items 5 and 6 on both scales. The former refers to the experience of fear without an apparent cause, while the latter addresses a sense of being overwhelmed by circumstances. Although these symptoms are common during the perinatal period, their broad and subjective wording may have led to varied interpretations among participants, as reflected in the inconsistency of their psychometric indicators. This variability may also stem from the fine line between expected emotional responses to a demanding life event and the presence of clinically significant distress. Accordingly, it would be advisable to reconsider the wording of these items in future versions of the instrument, exploring alternative formulations that offer greater precision and sensitivity in capturing variations within the construct being measured.

Regarding concurrent validity, both scales showed significant correlations with external measures related to anxiety, depression, and perceived stress, reinforcing their ability to capture critical emotional dimensions during this period. The PAPA scale showed weaker correlations than the PAMA scale with measures of depression and perceived stress, which may reflect gender differences in how men and women perceive, experience, and report emotional distress during the perinatal period. The highest correlations were observed with dimensions of Anxiety and Depression, measured by SCL-90 and EPDS (ρ between 0.54 and 0.69), highlighting the scales’ capacity to capture fundamental emotional constructs. Moderate correlations with measures such as Total Positive Symptoms (ρ between 0.35 and 0.44) and Somatization (ρ = 0.54) support the scales'applicability in evaluating psychophysiological aspects. Negative correlations with relational variables, such as Dyadic Cohesion and Consensus (ρ between −0.07 and −0.34), suggest a complex relationship between perinatal affectivity and couple dynamics, warranting further exploration in future research.

The differences in average scores between the PAPA and PAMA scales highlight a key finding: fathers tend to report higher levels of perinatal symptomatology than mothers, as reflected in higher scores on the PAPA scale. This result underscores the need to develop gender-specific instruments, considering differences in the expression of perinatal affective symptomatology. These differences may be related to psychosocial factors shaping how men experience and report affectivity, emphasizing externalizing symptoms as a typical male manifestation of emotional challenges during the perinatal period.

This study presents several limitations. First, the sample size was relatively small, which may affect the generalizability of the findings to the broader population. Second, the sample was skewed toward participants with higher educational levels, which may be indicative of a higher socioeconomic status. This limits the representativeness of the findings, particularly for populations at greater psychosocial risk. Another important limitation is the cross-sectional nature of the study, which prevents the assessment of the scales’ temporal stability. Although internal consistency was examined, the absence of follow-up data limits conclusions about the instruments’ reliability over time. Future research should incorporate longitudinal designs to evaluate the stability and predictive validity of the scales across different stages of the perinatal period. Also, future research should aim to recruit larger and more socioeconomically diverse samples, particularly including individuals from lower-income backgrounds, who are often more vulnerable to perinatal affective symptoms. In addition, upcoming studies should refine the structure of items that showed suboptimal performance, expand the sample's demographic variability.

## Conclusion

This study provides relevant evidence for the validation of the PAPA and PAMA scales, emphasizing their utility as tools for evaluating perinatal affectivity in clinical and population contexts in Chile. Overall, these results support the use of PAPA and PAMA as reliable and valid instruments for assessing relevant psychological constructs. Additionally, the findings highlight the importance of including fathers in perinatal mental health evaluations, promoting a dyadic perspective that considers the mutual influence of affective states between fathers and mothers during this critical period.

The present findings have relevant clinical and policy implications. Given their brevity, ease of administration, and gender-sensitive design, the PAPA and PAMA scales could be effectively integrated into routine perinatal care within primary healthcare settings. Their use during prenatal checkups or postnatal visits would enable early detection of emotional distress in both mothers and fathers, including symptoms that may not be captured by traditional screening tools. Implementing these instruments as part of standardized mental health protocols could support timely psychological referral and intervention, especially in populations that tend to underreport affective symptoms. This approach aligns with current recommendations to promote comprehensive and inclusive perinatal mental health screening practices.

## Data Availability

The datasets generated and analyzed during the current study are not publicly available due to [privacy concerns], but are available from the corresponding author on reasonable request.
